# A comprehensive analysis of *NDST3* for schizophrenia and bipolar disorder in Han Chinese

**DOI:** 10.1038/tp.2015.199

**Published:** 2016-01-05

**Authors:** C Zhang, W Lu, Z Wang, J Ni, J Zhang, W Tang, Y Fang

**Affiliations:** 1 Schizophrenia Program, Shanghai Mental Health Center, Shanghai Jiao Tong University School of Medicine, Shanghai, China; 2 Department of Psychiatry, Hongkou Mental Health Center of Shanghai, Shanghai, China; 3 Department of Psychiatry, Tongde Hospital of Zhejiang Province, Hangzhou, China; 4 Department of Psychiatry, Hangzhou Seventh People's Hospital, Hangzhou, China; 5 Division of Mood Disorders, Shanghai Mental Health Center, Shanghai Jiao Tong University School of Medicine, Shanghai, China

## Abstract

A novel susceptibility locus (rs11098403) for schizophrenia and bipolar disorder (BD) was identified in an Ashkenazi Jewish population by a recent large-scale genome-wide association study. The rs11098403 is located in the vicinity of the gene encoding *N*-deacetylase/*N*-sulfotransferase (heparan glucosaminyl) 3, (*NDST3*). This study aimed to replicate the results in a Han Chinese population and then potentially extend these findings. We performed a two-stage study to investigate the association of *NDST3* with the schizophrenia and BD risk in the Han Chinese. In stage 1, a total of 632 patients with schizophrenia, 654 patients with BD and 684 healthy controls were recruited from the Shanghai region. In stage 2, 522 schizophrenia patients and 547 normal subjects were enrolled from the Hangzhou region. Then, we conducted a meta-analysis based on the present literature. In stage 1, the single nucleotide polymorphism (SNP) rs11098403 showed a significant association with schizophrenia (corrected *P*=0.005). The frequency of the rs11098403 G allele was significantly lower among schizophrenia patients than among the controls (odds ratio (OR)=0.68, 95% confidence interval (CI): 0.55−0.84, corrected *P*=0.002). No significant difference was observed in individual SNP marker genotypes or allele distributions between the BD and control groups. In stage 2, the association of rs11098403 with schizophrenia could be validated (genotypic *P*=0.001 and allelic *P*=0.0003). After pooling all data from 1861 patients with schizophrenia and 2081 controls, we observed a significant association of the rs11098403 G allele with schizophrenia (*Z*=5.56, *P*<0.001), with an OR=0.70 (95% CI: 0.61−0.79). Then, we performed an expression quantitative trait loci analysis to investigate the functional effect of rs11098403 on *NDST3* expression in the brain. We observed a significant association of rs11098403 with *NDST3* expression in the hippocampus (*P*=0.027), although the significance did not survive after multiple testing correction. Our findings provided preliminary evidence that rs11098403 might modify the genetic risk of schizophrenia in the Han Chinese. Further investigations are warranted to identify the precise mechanism regulating brain *NDST3* expression in the Han Chinese. These results would help to explain the pathophysiological mechanism of schizophrenia.

## Introduction

Over the past 100 years, the functional psychoses have been divided into two major diagnostic categories: schizophrenia and bipolar disorder (BD).^1^ However, in clinical practice there is no sharp symptomatic distinction between the two neuropsychiatric diseases.^2^ Although their pathophysiological mechanisms are not clearly understood, there is compelling evidence from family, twin and adoption studies supporting the involvement of a genetic predisposition in schizophrenia and BD, with estimated heritability up to ~80% for both disorders.^3^ Some overlapping genetic influences between the two diseases have been reported in genome-wide linkage screenings.^4, 5^ Therefore, a specific genetic basis may be shared by schizophrenia and BD.

The genome-wide association study (GWAS) is currently the most powerful, systematic and unbiased genetic approach to identify susceptibility variants in complex disorders such as schizophrenia and BD.^6^ A novel susceptibility single nucleotide polymorphism (SNP; rs11098403) that emerged from a recent large-scale GWAS^7^ was demonstrated to have significant associations with schizophrenia and BD in an Ashkenazi Jewish population. The rs11098403 SNP is located within an intergenic region in the vicinity of the gene encoding *N*-deacetylase/*N*-sulfotransferase (heparan glucosaminyl) 3, (*NDST3*); the 5′ end of this gene is ~308 kb from this locus.^7^ *NDST3* is expressed abundantly in the hippocampus and cerebellum,^8^ in which structural and functional abnormalities are consistently observed in patients with schizophrenia^9, 10^ and BD.^11^ Therefore, this GWAS provided suggestive evidence that the *NDST3* variation predisposed patients to schizophrenia and BD.

Poor replication is an important issue in genetic association studies. Therefore, these studies require cautious replication validation.^12^ In a recent replication study, rs11098403 demonstrated a nominally significant association with schizophrenia in a Han Chinese population.^13^ However, the small sample size limited the ability to draw any definitive conclusion due to the low statistical power of detection coinciding with increased rates of both false positives and false negatives.^14^ In this study, we first aimed to replicate the association of rs1109803 with schizophrenia and BD in Han Chinese samples from the Shanghai region. Our results replicated the positive association between rs1109803 and schizophrenia. Subsequently, we validated this positive result in an independent Chinese Han population from the Hangzhou region. To overcome the limitation of the small sample size, we performed a meta-analysis to clarify the association between rs1109803 and schizophrenia in the Han Chinese. Second, although the GWAS data indicated that the *NDST3* variation possibly predisposed patients to schizophrenia and BD, to the best of our knowledge no genetic study has identified an association of *NDST3* with schizophrenia and BD. Therefore, we performed an association study to address this issue. Third, a prior literature search provided evidence that the intronic SNP rs11098403 had a regulatory effect on the expression of *NDST3* in postmortem cerebellar tissue.^7^ We validated the role of rs11098403 in *NDST3* expression in the brain using expression quantitative trait loci (eQTL) analysis via an available database.

## Materials and methods

### Subjects

All subjects were of Han Chinese descent and provided written informed consent before the performance of any procedures related to this study. All procedures of this study were reviewed and approved by the Institutional Review Boards of the Shanghai Mental Health Center and local institutions. This study was performed in strict accordance with the Declaration of Helsinki and other relevant national and international regulations.

We performed this study using a multi-stage association design. For the first stage, 632 patients with schizophrenia and 654 patients with BD were collected from the Shanghai Mental Health Center, Shanghai Jiao Tong University School of Medicine. Six hundred and eighty-four healthy controls were recruited from the hospital staff and students of the School of Medicine in Shanghai and then interviewed by a specialized psychiatrist using the Structured Clinical Interview for DSM-IV-TR Axis I Disorders-Patient Edition. Stage 2 included 522 schizophrenia patients and 547 normal subjects from the Hangzhou region. The patients were enrolled from the Tongde Hospital of Zhejiang Province and the Hangzhou Seventh People's Hospital. The controls were randomly selected from the general populations in Hangzhou. The demographics of the analyzed populations were presented in Supplementary Table S1.

The patients with schizophrenia or BD were recruited on the basis of previously established criteria.^14, 15, 16, 17, 18^ Diagnosis and review of psychiatric case records were independently checked and verified by two experienced psychiatrists to ensure consistency. None of the control subjects had any prior or current psychiatric disorders and/or chronic physical diseases.

### SNP selection and genotyping

The principal hypothesis underlying this study is that common SNPs in *NDST3* may confer susceptibility to schizophrenia. In total, five selected tagging SNPs of *NDST3* were genotyped to test for a possible association. To set inclusion criteria for tagging SNPs, we retrieved CHB data from the HapMap database (http://www.hapmap.org) and defined linkage disequilibrium (LD) blocks using Haploview 4.2 (Broad Institute, Cambridge, MA, USA). Haplotype-tagging SNPs (htSNPs) were selected with an *r*^2^ cutoff >0.8 and minor allele frequency >0.2, resulting in the capture of five htSNPs (rs6534078, rs2389519, rs12642204, rs4327555 and rs631271). The detailed information of the selected SNPs was presented in Supplementary Table S2. Genomic DNA of all participants was extracted from the peripheral blood using a Tiangen DNA Isolation Kit (Tiangen Biotech, Beijing, China). The five htSNPs were amplified independently via PCR and genotyped by direct sequencing using an ABI PRISM 3730 Genetic Analyzer (Perkin-Elmer Applied Biosystems, Foster City, CA, USA). Genotyping was performed according to the methods described in our previous studies.^14, 15^ PCR amplification was performed in a volume of 25 μl containing the primer pair for each SNP. PCR primers were also used for sequencing. Sequencing results were analyzed with DNAStar (DNAStar, Madison, WI, USA), and the original sequencing chromatograms of each sample were manually checked.

Quality control methods were based on our previous work.^16, 19, 20^ Ten percent of the samples were randomly selected and independently sequenced to evaluate the genotype accuracy. Here our re-sampling results were 99.91% consistent with the genotype data from the original analysis.

### Meta-analysis

The meta-analysis was performed in agreement with previously described methods.^14^ The literature included in the analysis was selected using PubMed (http://www.ncbi.nlm.nih.gov/pubmed/) and SCOPUS (http://www.scopus.com) searching the keywords ‘NDST3 or rs11098403', ‘Han Chinese', and ‘schizophrenia, SZ or SCZ' in varying combinations. Bibliographies or citations from the retrieved articles were also checked. Reports from these searches included research published up to June, 2015. Eligible studies were included in our meta-analysis if they met all of the following criteria: (1) association studies written in English or Chinese; (2) described the genotyping method and used commonly acceptable diagnosis criteria, such as the DSM; (3) contained independent data; and (4) presented sufficient data to calculate the odds ratio (OR) with a confidence interval (CI) and *P*-value. The major exclusion criteria included deviation from the Hardy−Weinberg equilibrium, overlap with previous studies, or insufficient information for extraction. The authors were contacted in cases where there were queries regarding their studies. Two independent authors extracted the following data from each eligible study: last name of the first author, year of publication, and allele frequencies of cases and controls.

### Brain eQTL analysis

Considering that mental illness reasonably originates from abnormal brain functions, brain samples are presumably appropriate for eQTL analysis of risk SNP(s).^17^ Hence, we used the brain eQTL database (http://caprica.genetics.kcl.ac.uk/BRAINEAC/), which is a large exon-specific eQTL data set covering ten human brain regions, for this analysis. The BRAINEAC database is based on 134 healthy Caucasian brain samples. More detailed information can be found in the original study.^21^

### Statistical analysis

Hardy−Weinberg equilibrium testing and allele and genotype frequency analysis were conducted using SHEsis (http://analysis.bio-x.cn).^22^ Pairwise LD of all pairs of htSNPs was assessed using HaploView 4.2 (Broad Institute),^23^ and the extent of LD was measured by the standardized *D*′ and *r*^2^. Power calculations were performed with Quanto 1.2.3 (http://hydra.usc.edu/GxE). To rule out population stratification between the two disorders and control populations, additional genotyping data for the six selected SNPs in this study were analyzed with the STRUCTURE software (version 2.3.4, http://pritch.bsd.uchicago.edu/structure.html). To adjust for multiple testing, the level of significance was corrected via Bonferroni correction. For the meta-analysis, data were summarized using two-by-two tables. The Cochran *χ*^2^-based Q statistical test was performed to assess heterogeneity and ensure that each group of studies was suitable for meta-analysis. ORs were pooled using the methods described by DerSimonian,^24^ and 95% CIs were constructed using Woolf's method.^25^ If the result of the heterogeneity test was *P*>0.05, the ORs were pooled according to the fixed-effects model (Mantel−Haenszel methods); otherwise, the random-effects model was used. Funnel plots of the studies' effect estimates against sample size were used to validate the meta-analysis.^26^ The above statistical analyses were performed using STATA 12.0 (Stata, College Station, TX, USA). The criterion for statistical significance was set at *α*=0.05, and all values were two-tailed.

## Results

The genotype distributions of the studied SNPs in the control group were in accordance with the Hardy−Weinberg equilibrium. The results of single-marker analyses in stage 1 were shown in Table 1. The SNP rs11098403 showed a significant association with schizophrenia (*P*=0.0009, *P*=0.005 following Bonferroni correction). The frequency of the G allele of rs11098403 was significantly lower among schizophrenia patients than among the controls (OR=0.68, 95% CI: 0.55−0.84, *P*=0.0004, *P*=0.002 after Bonferroni correction). There were no significant differences in genotype or allele distributions of the tagSNPs within *NDST3* between the schizophrenia and control groups. Analysis of pairwise LD was performed in SNP pairs. We observed two strong pairwise LD blocks in the schizophrenia samples (Supplementary Figure S1), and a multimarker haplotype analysis was performed. Supplementary Table S3 listed all *P*-values corresponding to the haplotypes, with rare haplotypes (<3%) being dropped. We found no significant association between the haplotypes and schizophrenia. In a comparison between the BD and control groups, no significant difference was observed in individual SNP marker genotypes or allele distributions (Table 1). Because strong pairwise LDs were found between all studied SNPs in the BD samples (Supplementary Figure S1), we also performed haplotype analysis for the six SNPs. Again, no significant haplotype difference was observed (Supplementary Table S4). In stage 2, the association of rs11098403 with schizophrenia could be validated in the samples from the Hangzhou region (genotypic *P*=0.001 and allelic *P*=0.0003, Table 2). With the false-positive rate controlled at 0.05, the statistical power to detect the OR value as 1.5 for the risk allele was expected to be >90% in each SNP analysis under a log additive model. Supplementary Figure S2 presented the triangle charts of the results of the population stratification analysis in schizophrenia, BD and control populations, respectively. Our results showed that the schizophrenia, BD and control samples spattered evenly within the triangle charts, which denied the distinct significant stratification in the population.

**Figure 1:**
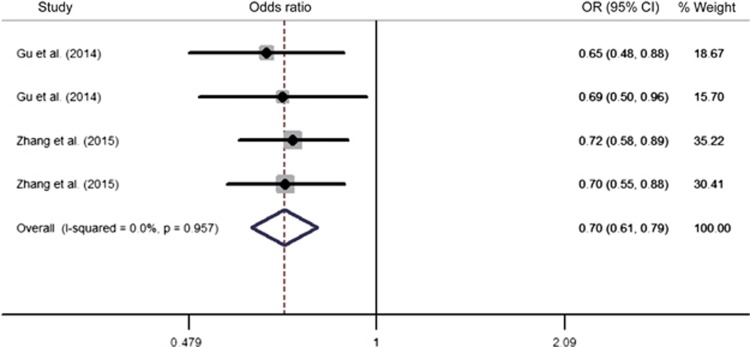
Meta-analysis between rs11098403 and schizophrenia in the Han Chinese. ORs and 95% CIs of individual studies and pooled results for all included studies between the G allele of the rs11098403 polymorphism and schizophrenia. The sources of the published data were listed in Supplementary Table S5.

To obtain a more comprehensive view of the association of rs11098403 with schizophrenia in the Han Chinese, a meta-analysis was conducted by pooling previous and the present data. Four sample populations contributed to this meta-analysis. There was no evidence of significant between study heterogeneity (*I*^2^=0.0%, *P*=0.96). We subsequently performed a meta-analysis using the fixed-effects method. After pooling all data from the 1861 patients with schizophrenia and the 2081 controls, we observed a significant association of the rs11098403 G allele with schizophrenia (*Z*=5.56, *P*<0.001), with an OR=0.70 (95% CI: 0.61−0.79) (Figure 1). There was no evidence of publication bias in the meta-analysis by the trim and fill method. The detailed information of the rs11098403 G allele frequency for each population was described in Supplementary Table S5.

**Figure 2:**
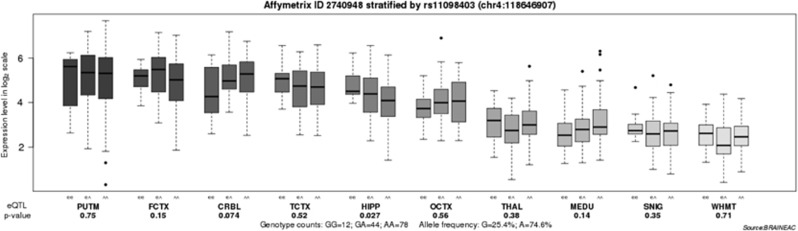
Association of rs11098403 with the *NDST3* mRNA expression level in ten brain regions (Affymetrix ID 2740948). CRBL, cerebellar cortex; FCTX, frontal cortex; HIPP, hippocampus; MEDU, the inferior olivary nucleus (sub-dissected from the medulla); OCTX, occipital cortex; PUTM, putamen (at the level of the anterior commissure); SNIG, substantia nigra; TCTX, temporal cortex; THAL, thalamus (at the level of the lateral geniculate nucleus); WHMT, intralobular white matter. Data were extracted from the BRAINEAC database (http://caprica.genetics.kcl.ac.uk/BRAINEAC/).

Then, we performed an eQTL analysis to investigate the functional effect of rs11098403 on *NDST3* expression in the brain. There were 19 exon-level and 1 gene-level *NDST3* transcripts in the BRAINEAC database. As shown in Figure 2, there was a significant association of rs11098403 with *NDST3* expression in the hippocampus (*P*=0.027), but this significance did not survive after Bonferroni correction. Compared with the A allele of rs11098403, the G allele showed a higher level of *NDST3* expression in the hippocampus.

## Discussion

Recently, *NDST3* was implicated as a major factor in both schizophrenia and BD pathogenesis; this finding was supported by a GWAS analysis. In this study, we performed a comprehensive investigation of the association of *NDST3* with schizophrenia and BD in the Han Chinese. Both our initial results and subsequent meta-analysis replicated the earlier GWAS showing a genetic association between the SNP rs11098403 with schizophrenia in the Han Chinese.

In our control subjects, the frequency of the rs11098403 G allele was 17.7%, which was similar to the frequency in the Han Chinese from the HapMap database (frequency=17.4%). Our meta-analysis showed that the OR of the rs11098403 G allele for schizophrenia in the Han Chinese was 0.70 and the 95% CI was from 0.61 to 0.79. However, we noted that the significance of the rs11098403 G allele in schizophrenia appeared in Caucasian populations with OR=1.41 (*P*=6.55 × 10^9^; ref. 7) and OR=1.21 (*P*=0.03).^27^ The above findings implied that the rs11098403 G allele was likely to be a protective factor for schizophrenia in the Han Chinese and a risk factor for schizophrenia in Caucasians. To clarify the effect of rs11098403 on *NDST3* expression in the brain, we performed an eQTL analysis and observed that rs11098403 might have a regulatory effect on *NDST3* expression in the hippocampus; additionally, we found that the G allele resulted in a higher level of *NDST3* expression than the A allele. The SNP rs11098403 is located in an intragenic region in the vicinity of *NDST3*, although our data showed that there was no LD between rs11098403 and the nearby loci within *NDST3*. NDST3 is an enzyme that is expressed in both the fetal and adult brain, with the highest abundance in the cerebellum and hippocampus.^8^ This enzyme has a critical role in maintaining heparan sulfate metabolism, and knockout of *NDST3* in mice leads to a substantial reduction of heparan sulfate sulfation and affects the physiological functions in the brain.^28^ There is a growing body of evidence showing that the hippocampus is one of the most significant areas in the pathophysiology of schizophrenia.^29^ Structural and functional abnormalities in the hippocampus have been frequently reported in patients with schizophrenia. A recent large-scale magnetic resonance imaging data analysis showed that patients with schizophrenia had small hippocampi compared with healthy controls and that hippocampal volume deficits were significantly associated with a high proportion of unmedicated patients.^30^ The aberrant hippocampal volume could be partially ameliorated by atypical antipsychotic treatment.^31^ Allen *et al.*^32^ measured adult hippocampal neurogenesis in patients with schizophrenia and observed that the cell proliferation marker Ki67 was significantly reduced in their postmortem hippocampal tissues. Taken together, these results indicate that the G allele of rs11098403 is associated with a higher level of *NDST3* expression, allowing NDST3 to maintain the normal neurodevelopmental processes in the hippocampus and thereby have a protective role in the development of schizophrenia.

In this study, we did not replicate the significance of *NDST3* with BD reported in Caucasian populations.^7^ This result is in line with previous GWAS findings conducted in a Taiwan Han Chinese population.^33^ In clinical practice and genetic studies, both symptomatic and genetic overlaps between BD and schizophrenia have been noted.^17, 34^ An epidemiological investigation indicated that a high prevalence of psychotic symptoms was observed in patients with BD-I.^35^ Thus, investigating BD by subtype category could yield greater power in genetic studies.^36^ Although the current literature did not support the hypothesis that *NDST3* conferred susceptibility to BD in the Han Chinese, further work with a larger sample size and rigorous study design is required to better understand the relationship between *NDST3* and BD.

Although our control samples were ethnically matched to the case subjects and the minor allele frequency of each SNP was similar to that in the CHB from the HapMap database, case-control association analyses always have potential biases for population stratification. Ample evidence suggests that population stratification exists within the Han Chinese population.^37, 38^ Therefore, we performed a population stratification analysis to exclude the possibility of our results occurring as a result of population stratification. Here we did not observe distinct population stratification in our samples. This result suggested that our outcomes were unlikely to be the consequences of potential population stratification.^39^ Transmission/disequilibrium tests help to avoid population stratification.^40^ Therefore, further family based studies are required to validate our current findings.

Several limitations in this study should be noted. First, our eQTL analysis was performed in the BRAINEAC database derived from Caucasian brain samples. It is unclear whether the modulatory effect of the rs11098403 polymorphism affects hippocampal *NDST3* expression in the Han Chinese. Thus, our findings need to be considered as preliminary and exploratory. Second, the size of our sample is modest. To avoid the possibility of reporting type II errors, more replication and independent verification are required. Third, only data from published studies was included in our meta-analysis, while other techniques (for example, subgroup analysis and meta-regression) were not included because not enough information was available to reliably include these results in our larger analysis.

In conclusion, we performed a comprehensive analysis to replicate the early GWAS results in Chinese Han populations. Our findings provided preliminary evidence that rs11098403 might modify the genetic risk of schizophrenia in the Han Chinese. Further investigations are warranted to validate our findings and identify the precise mechanism by which rs11098403 regulates brain *NDST3* expression in the Han Chinese. These results would help to explain the pathophysiological mechanism of schizophrenia.

## Figures and Tables

**Table 1:** Genotypic and allelic distributions of the selected SNPs in patients with schizophrenia, BD and healthy controls in Shanghai sample

*SNP*	*Sample*	N	*Genotype*, n *(%)*			P*-value*^a^	P-*value*^b^	*Allele*, n *(%)*		*OR (95% CI)*	P*-value*^a^	P*-value*^b^	P*-value*^c^
rs11098403			G/G	G/A	A/A			G	A				
	Schizophrenia	632	5 (0.8)	151 (23.9)	476 (75.3)	0.0009	**0.005**	161 (12.7)	1103 (87.3)	0.68 (0.55−0.84)	0.0004	**0.002**	
	BD	654	15 ((2.3)	162 (24.8)	477 (72.9)	0.08		192 (14.7)	1116 (85.3)	0.80 (0.65−0.98)	0.03	0.18	
	Control	684	18 (2.6)	206 (30.1)	460 (67.3)			242 (17.7)	1126 (82.3)				0.37
													
rs6534078			A/A	A/G	G/G			A	G				
	Schizophrenia	632	40 (6.3)	230 (36.4)	362 (57.3)	0.06		310 (24.5)	954 (75.5)	0.84 (0.71−1.01)	0.06		
	BD	654	46 (7.0)	240 (36.7)	368 (56.3)	0.08		332 (25.4)	976 (74.6)	0.88 (0.74−1.05)	0.16		
	Control	684	44 (6.4)	292 (42.7)	348 (50.9)			380 (27.8)	988 (72.2)				0.09
													
rs2389519			T/T	T/G	G/G			T	G				
	Schizophrenia	632	22 (3.5)	204 (32.3)	406 (64.2)	0.33		248 (19.6)	1016 (80.4)	0.89 (0.73−1.07)	0.22		
	BD	654	29 (4.4)	189 (28.9)	436 (66.7)	0.02	0.12	247 (18.9)	1061 (81.1)	0.85 (0.70−1.02)	0.08		
	Control	684	24 (3.5)	247 (36.1)	413 (60.4)			295 (21.6)	1073 (78.4)				0.08
													
rs12642204			T/T	T/C	C/C			T	C				
	Schizophrenia	632	43 (6.8)	227 (35.9)	362 (57.3)	0.14		313 (24.8)	951 (75.2)	0.89 (0.75−1.06)	0.18		
	BD	654	44 (6.7)	228 (34.9)	382 (58.4)	0.05	0.30	316 (24.2)	992 (75.8)	0.86 (0.72−1.02)	0.09		
	Control	684	44 (6.4)	282 (41.2)	358 (52.3)			370 (27.0)	998 (73.0)				0.24
													
rs4327555			T/T	T/A	A/A			T	A				
	Schizophrenia	632	117 (18.5)	317 (50.2)	198 (31.3)	0.05	0.30	551 (43.6)	713 (56.4)	0.83 (0.71−0.97)	0.02	0.12	
	BD	654	148 (22.6)	308 (47.1)	198 (30.3)	0.24		604 (46.2)	704 (53.8)	0.92 (0.79−1.07)	0.28		
	Control	684	156 (22.8)	348 (50.9)	180 (26.3)			660 (48.2)	708 (51.8)				0.62
													
rs631271			A/A	A/G	G/G			A	G				
	Schizophrenia	632	31 (4.9)	178 (28.2)	423 (66.9)	0.03	0.18	240 (19.0)	1024 (81.0)	0.85 (0.70−1.02)	0.08		
	BD	654	18 (2.8)	244 (37.3)	392 (59.9)	0.26		280 (21.4)	1028 (78.6)	0.98 (0.82−1.18)	0.85		
	Control	684	29 (4.2)	239 (34.9)	416 (60.8)			297 (21.7)	1071 (78.3)				0.47

Abbreviations: BD, bipolar disorder; CI, confirdence interval; N, number of samples; n, number of genotypes and alleles; OR, odds ratio.

Significances after Bonferroni correction were highlighted in bold.

a The *P*-values for raw data.

b The *P*-values were adjusted by Bonferroni correction.

c The *P*-values for Hardy−Weinberg equilibrium.

**Table 2:** Validation of the association between rs11098403 and schizophrenia in Hangzhou sample

	*Sample*	N	*Genotype*, n *(%)*			P*-value*	*Allele*, n *(%)*		P*-value*	*OR (95%CI)*
rs11098403			G/G	G/A	A/A		G	A		
	Cases	522	7 (1.3)	124 (23.8)	391 (74.9)	0.001	138 (13.2)	906 (86.8)	0.0003	0.65 (0.52−0.83)
	Controls	547	13 (2.4)	181 (33.1)	353 (64.5)		207 (18.9)	887 (81.1)		

Abbreviations: CI, confirdence interval; N, number of samples; n, number of genotypes and alleles; OR, odds ratio.
