# Supplementary material for: A comprehensive analysis of NDST3 for schizophrenia and bipolar disorder in Han Chinese
Source: Transl Psychiatry. 2016 Jan 5;6(1):e701–. doi: 10.1038/tp.2015.199 (PMC5068873; doi:10.1038/tp.2015.199)

Supplementary Table S1 Demographics of the analyzed populations

|  | Stage-1 | | |  | Stage-2 | |
| --- | --- | --- | --- | --- | --- | --- |
|  | SCZ | BD | CTL |  | SCZ | CTL |
| Number of subjects (n) | 632 | 654 | 684 |  | 522 | 547 |
| Age (years), mean (SD) | 34.2 (9.4) | 35.3 (10.2) | 33.8 (7.1) |  | 33.7 (5.9) | 34.4 (7.6) |
| Gender, male n (%) | 358 (56.7) | 292 (44.6) | 327 (47.8) |  | 316 (60.5) | 332 (60.7) |

SCZ, schizophrenia; BD, bipolar disorder; CTL, control

Supplementary Table S2 Information of selected SNPs genotyped in this study

| # | SNP ID | Allele | MAF ^a^ | Position (Chr. 4) | Note |
| --- | --- | --- | --- | --- | --- |
| 1 | rs11098403 | A/G | 0.174 | 118646907 | Functional variant |
| 2 | rs6534078 | A/G | 0.221 | 118953901 | Tag |
| 3 | rs2389519 | G/T | 0.186 | 119009752 | Tag |
| 4 | rs12642204 | C/T | 0.268 | 119064257 | Tag |
| 5 | rs4327555 | A/T | 0.442 | 119170929 | Tag |
| 6 | rs631271 | A/G | 0.214 | 119175904 | Tag |

**^a^** MAF - minor allele frequency, in dbSNP CHB dataset (<http://www.ncbi.nlm.nih.gov/SNP/>).

Supplementary Table S3 Haplotype analysis of *NDST3* in schizophrenia and control groups

| Haplotype^a^ | Frequency (%) | | | χ^2^ | *P*^c^ | *P*^d^ |
| --- | --- | --- | --- | --- | --- | --- |
|  | Total | Cases | Controls |  |  |  |
| G-G | 72.9 | 74.2 | 71.6 | 2.23 | 0.14 |  |
| A-T | 19.7 | 18.4 | 21.0 | 2.82 | 0.09 |  |
| A-G | 6.5 | 6.1 | 6.8 | 0.45 | 0.50 |  |
| Haplotype^b^ |  |  |  |  |  |  |
| C-A-G | 53.4 | 55.9 | 51.2 | 5.83 | 0.02 | 0.08 |
| T-T-G | 25.3 | 23.9 | 26.6 | 2.65 | 0.10 |  |
| C-T-A | 19.8 | 18.5 | 21.1 | 2.75 | 0.10 |  |

^a^ rs6534078-rs2389519 haplotypes

^b^ rs12642204-rs4327555- rs631271 haplotypes

^c^ The *P* values for raw data

^d^ The *P* values were adjusted by 1,000 permutation test

Haplotypes with frequency less than 0.03 are ignored in analysis

Supplementary Table S4 Haplotype analysis of *NDST3* in BD and control groups

| Haplotype^a^ | Frequency (%) | | | χ^2^ | *P*^c^ | *P*^d^ |
| --- | --- | --- | --- | --- | --- | --- |
|  | Total | Cases | Controls |  |  |  |
| G-G | 72.4 | 73.2 | 71.6 | 0.81 | 0.37 |  |
| A-T | 19.2 | 17.4 | 21.0 | 5.34 | 0.02 | 0.10 |
| A-G | 7.4 | 7.9 | 6.8 | 1.25 | 0.26 |  |
| Haplotype^b^ |  |  |  |  |  |  |
| C-A-G | 52.4 | 53.7 | 51.1 | 1.70 | 0.19 |  |
| T-T-G | 25.4 | 24.0 | 26.7 | 2.49 | 0.11 |  |
| C-T-A | 21.2 | 21.4 | 21.1 | 0.04 | 0.84 |  |

^a^ rs6534078-rs2389519 haplotypes

^b^ rs12642204-rs4327555- rs631271 haplotypes

^c^ The *P* values for raw data

^d^ The *P* values were adjusted by 1,000 permutation test

Haplotypes with frequency less than 0.03 are ignored in analysis

Supplementary Table S5 Meta-analysis for rs11098403

|  |  | Cases | | Controls | |  |
| --- | --- | --- | --- | --- | --- | --- |
| Author, Year | Sample area | Events | Total | Events | Total | OR (95%CI) |
| Gu et al, 2014 | Wuxi | 76 | 880 | 120 | 900 | 0.61 (0.45-0.83) |
| Gu et al, 2014 | Qingdao | 59 | 534 | 128 | 800 | 0.65 (0.47-0.91) |
| Zhang, et al, 2015 | Shanghai | 161 | 1264 | 242 | 1368 | 0.68 (0.55-0.84) |
| Zhang, et al, 2015 | Hangzhou | 138 | 1044 | 207 | 1094 | 0.65 (0.52-0.83) |

Supplementary Figure S1 Linkage disequilibrium plots consisting of 6 SNPs within *NDST3* in schizophrenia and bipolar disorder. Pairwise linkage disequilibrium (LD) was computed for all possible combinations using the values of *D*’ and *r*^2^. (A) schizophrenia; (B) bipolar disorder


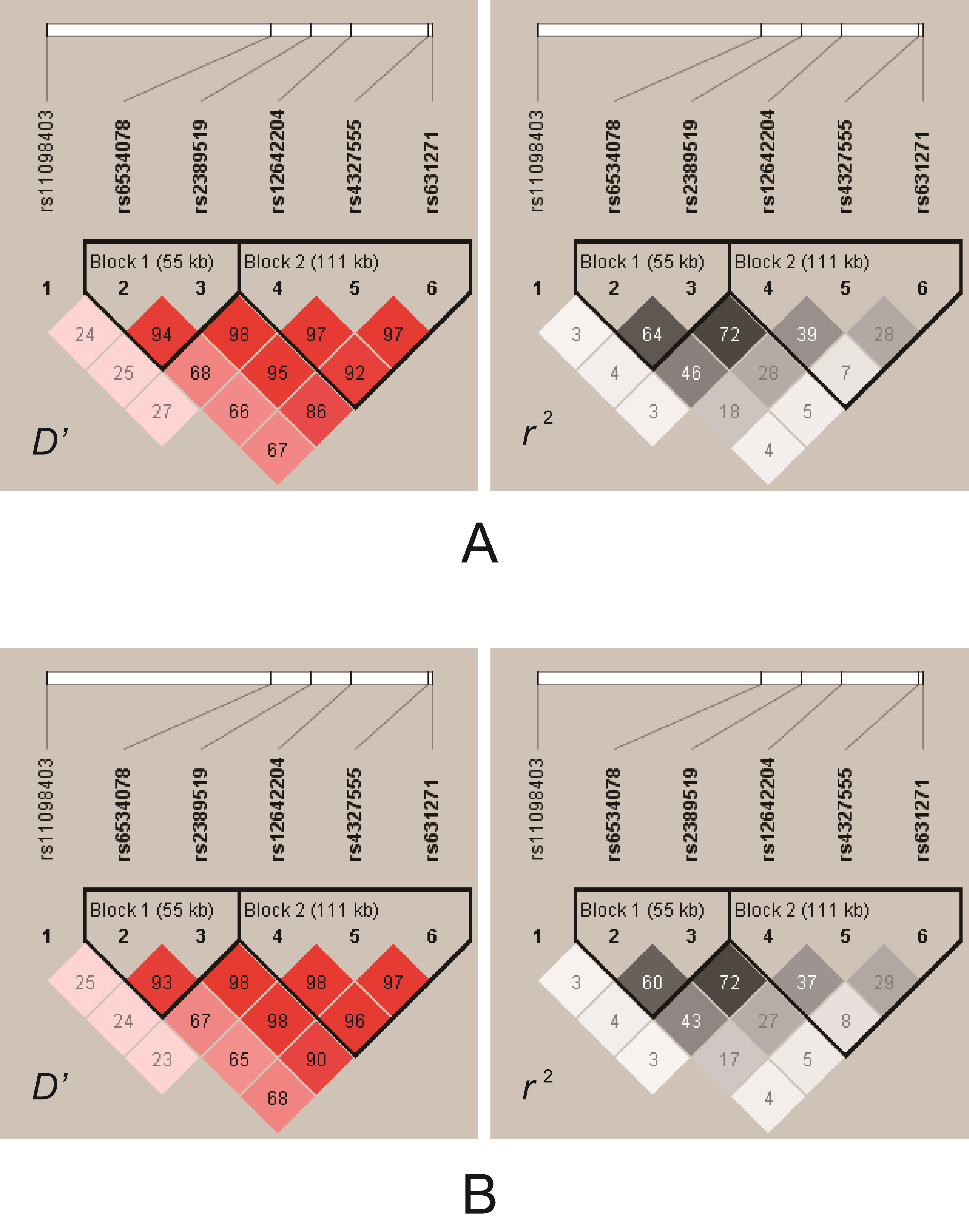


Supplementary Figure S2 Triangle charts towards population stratification analysis when *K*=3. Our samples from the two disorders and controls, in which SCZ represents schizophrenia, BD represents bipolar disorder and CTL represents healthy control populations, respectively. Allele-frequency divergence among populations computed using point estimates showed no evident admixture (*P*s>0.05, mean value of α=0.04). Therefore, the distributions of our samples of the two disorders and controls in the triangles indicate that there was no obvious population stratification in our samples.


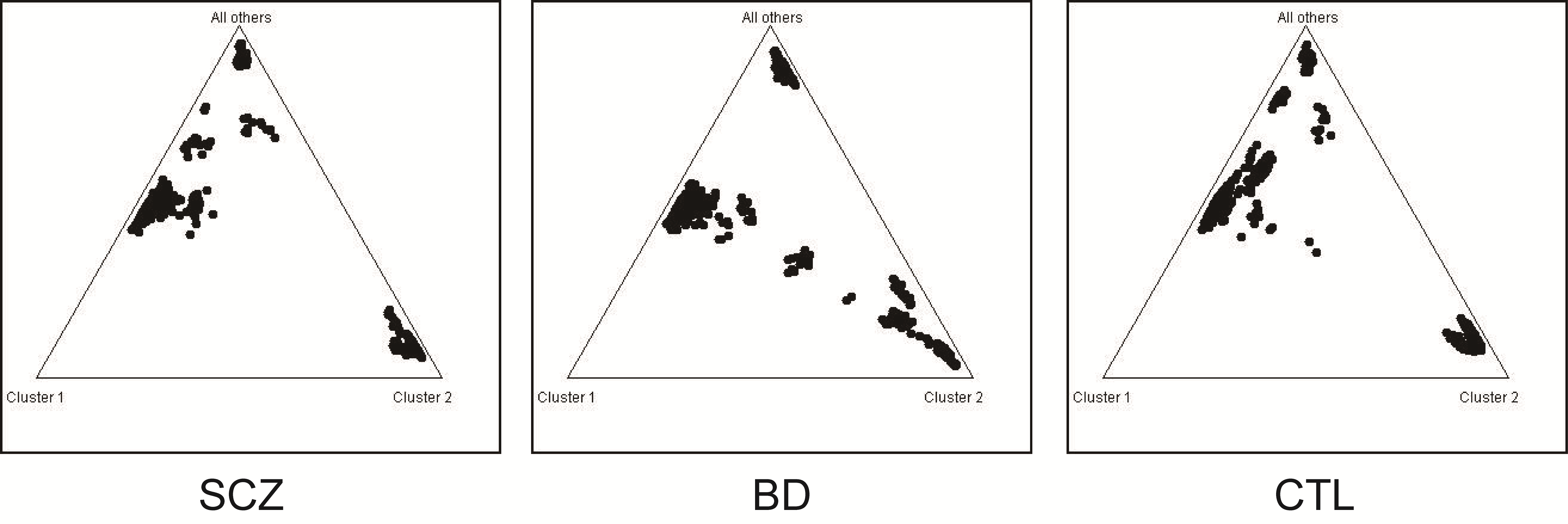

Supplement: Supplementary Information [file tp2015199x1.doc]
